# Immediate Transurethral Plasma Kinetic Enucleation of the Prostate Gland for Treatment of Benign Prostatic Hyperplasia-Associated Massive Hemorrhage: A Single-Center Experience

**DOI:** 10.3389/fsurg.2021.810175

**Published:** 2022-01-12

**Authors:** Yu Gan, Liang Deng, Qiangrong He, Chao Li, Leye He, Zhi Long

**Affiliations:** ^1^Department of Urology, Xiangya Hospital, Central South University, Changsha, China; ^2^Andrology Center, Department of Urology, The Third Xiangya Hospital, Central South University, Changsha, China

**Keywords:** benign prostatic hyperplasia, transurethral plasma kinetic enucleation of the prostate gland, hemorrhage, lower urinary tract symptoms, minimally invasive

## Abstract

**Purpose:** Benign prostatic hyperplasia-associated massive hemorrhage is a urological emergency. We evaluated the outcome from immediate transurethral plasma kinetic enucleation of the prostate gland (i-TUPKEP) for BHM treatment.

**Methods:** We retrospectively analyzed the records of 49 patients with acute BMH who underwent i-TUPKEP between January 2014 and November 2018 at our institution. The hemostatic effect, International Prostate Symptom Score (IPSS), and quality of life (QoL) score were evaluated preoperatively as well as 3, 6, and 12 months postoperatively. Postoperative follow-up also included measurement of the peak flow rate (Q_max_) and post-void residual urine volume (PVR). Clinical characteristics, weight of resected tissue, duration of bladder irrigation, duration of hospital stay, complications, as well as the time required for enucleation and resection, were recorded.

**Results:** BMH causes were attributed to transurethral surgery (17/49, 34.7%), violent catheterization (13/49, 26.5%), cystoscopy (10/49, 20.4%), and urethral dilatation (9/49, 18.4%). Bleeding was from different sites of prostate-gland tissues during i-TURKEP. i-TUPKEP-controlled BMH effectively induced immediate, notable, and lasting improvements in the IPSS and QoL score. Q_max_ was close to normal, and the PVR was within the physiological range, postoperatively. Long-term complications were not observed.

**Conclusion:** Our preliminary data suggest that i-TUPKEP is a feasible method for controlling BHM and relieving BPH symptoms.

## Introduction

Hematuria is a common complication of benign prostatic hyperplasia (BPH), which is often caused by injury to the prostatic urethra and gland (1). Although mild bleeding can be controlled through conservative treatment methods (2, 3), massive hemorrhage due to transurethral examination and/or surgery can develop into a urological emergency (4, 5). Rapid and extensive deposition of blood clots in the bladder can lead to catheter blockage, induction of bladder contractions, and aggravation of pain. Moreover, persistent blood loss without effective control can induce cardiovascular and cerebrovascular complications or lead to hemorrhagic shock and, sometimes, death (6, 7). Therefore, eliminating blood clots from the bladder and controlling BPH-associated massive hemorrhage (BMH) at the earliest opportunity is crucial.

In general, BMH indicates a need for surgical intervention. However, most patients with BPH patients are elderly and likely to have one or more diseases, so the risks of surgery and anesthesia increase exponentially (5, 8). Transurethral resection of the prostate gland (TURP) is a minimally invasive method to treat BMH. During TURP, blood clots can be cleared and the bleeding points can be found and treated by electrocoagulation. Nevertheless, good control of bleeding is a major challenge for the urologists (9). Some of the bleeding sites may be hidden in prostate-gland tissue, which will be shrunken and edematous. Moreover, residual prostatic tissue after TURP in this setting is likely to become a new bleeding site. In some cases, patients have to undergo another surgical procedure to stop the bleeding, which leads to an overall increased risk of complications (5, 6).

Transurethral plasma kinetic enucleation of the prostate gland combines the traits of a bipolar plasma kinetic device and enucleation of the prostate gland. TUPKEP has notably improved the efficacy and safety of transurethral surgical procedures for patients with BPH, especially with respect to bleeding control (10, 11).

Reports on TUPKEP application for BMH are rare. In consideration of the advantages of TUPKEP, we postulated that this method may be an alternative method for controlling BMH. After accumulating sufficient experience in using TUPKEP for BPH, we explored the efficacy and safety of TUPKEP for controlling BMH.

Our institution employs TUPKEP to eliminate blood clots and enucleate and resect the hyperplastic prostate gland in a one-stage emergency procedure for treating BMH, which is defined as “immediate TUPKEP” (i-TUPKEP). Here, we focused on evaluating the outcomes of i-TUPKEP for treating BMH.

## Materials and Methods

### Ethical Approval of the Study Protocol

The patients provided written informed consent before i-TURKEP. The study protocol was approved by the Ethics Review Board of Third Xiangya Hospital at Central South University (Changsha, China).

### Study Design

We retrospectively analyzed the records of 837 consecutive patients who underwent TUPKEP between January 2014 and November 2018 at Third Xiangya Hospital.

### Inclusion Criteria

The criteria for study inclusion were patients receiving TUPKEP for the following reasons: (i) BPH with active hemorrhage and extensive blood clots deposited in the bladder; (ii) continuous bladder irrigation was obstructed frequently and failed to alleviate pain and bladder contractions; (iii) BMH occurred immediately after inappropriate transurethral surgery or actions (e.g., repeat violent catheterizations, urethral dilatation, and cystoscopy).

### Exclusion Criteria

The exclusion criteria were a history of: (i) carcinoma in the prostate gland; (ii) taking anticoagulants or coagulation disorders in the perioperative period; (iii) myocardial infarction, cerebral infarction, or cerebral hemorrhage within 3 months before TUPKEP; (iv) neurogenic bladder, urethral stricture, or surgery of the lower urinary tract.

### Assessment Parameters

The patients underwent tests for the prostate-specific antigen (PSA) level; the hemoglobin level; serum levels of electrolytes, glucose, lipids, myocardial enzymes, brain natriuretic peptide, D-dimer; coagulation function; and liver and kidney functions. After hospital admission, blood-pressure measurement, electrocardiography, chest radiography, abdominal ultrasonography, and computed tomography were also conducted. Lower urinary tract symptoms (LUTS) were evaluated by the International Prostate Symptom Score (IPSS) and the Quality of Life (QoL) score based on status over the previous month before hospital admission. The prostate volume was assessed using transrectal ultrasonography (12). Owing to indwelling catheters, the patients did not undergo testing of the urinary flow rate or post-void residual urine volume (PVR) before surgery.

### Preoperative Treatments

Intravenous access was established rapidly. Central venous catheterization was undertaken if the patient had complications related to anemia and reduced blood pressure. Fluid rehydration and blood transfusion were carried out, and vasoactive drugs were administered (if necessary) based on the patient's condition. The preoperative blood pressure was maintained above 100/60 mmHg. All the patients were given antibiotics 30 min before the surgical procedure and received general anesthesia. All surgical procedures were undertaken by the same senior surgeon, who had >10 years of extensive experience in carrying out minimally invasive surgical procedures for BPH.

### Instruments and i-TUPKEP Procedure

An isoionic bipolar electrical cutting system (Gyrus Medical, Cardiff, Wales) used 200 W and 100 W for cutting and coagulation, respectively. Physiologic (0.9%) saline served as the irrigation fluid. A 27-Fr resectoscope with a loop was placed in the bladder through video-assisted endoscopic guidance system. Initially, blood clots were removed from the bladder using an Ellik evacuator. If removal of blood clots by the Ellik evacuator was difficult, the loop could be used for cutting them into small pieces. Overall observation of the urethra, verumontanum, prostatic urethra, bladder, and bilateral ureter orifices was done to determine the bleeding site. The main method of TUPKEP was based on the experience described by Xu and colleagues (13). The improvement we made was that the hyperplastic gland in the 12 o'clock position was resected in a retrograde fashion from the apex toward the bladder neck by the loop electrode instead of enucleation. A small and thin “pad” of the gland located between the 12 o'clock position of the urethral glands and apex was retained to improve urinary continence.

### Definition of “Surgical Success” and Postoperative Treatments

The success of the surgical procedure was defined as no-active bleeding after the surgical procedure. An indwelling, standard 20-F three-way catheter was used after the procedure, which was connected with a pump containing.9% saline for continuous irrigation of the bladder. Drainage was halted until hematuria had resolved sufficiently. As a routine procedure, the catheter was extracted 5 days after completion of the surgical procedure.

### Follow-Up

The patients were evaluated using the IPSS, QoL score, PSA level, hemoglobin level, blood pressure, the weight of all the prostatic fragments (in g), as well as the time needed for enucleation and resection and postoperative bladder irrigation during the perioperative period. Follow-up was conducted at 3, 6, and 12 months after surgery, which included assessment of the IPSS, QoL score, maximum urine flow rate (Q_max_) and the PVR. Postoperative complications were classified using the Clavien–Dindo system (14).

### Statistical Analyses

Data are the median, with minima and maxima. Mann–Whitney U test was used to analyze the differences between two independent samples, while Wilcoxon signed-rank test was used to analyze the differences between two related samples. Statistical analyses were conducted using SPSS 20.0 (IBM, Armonk, NY, USA). *p* < 0.05 was considered significant.

## Results

### Bleeding Causes

Of the 837 participants, 49 patients satisfied the inclusion criteria mentioned above. Table 1 outlines the baseline demographic data (including vital signs, anemia severity, and LUTS). The major cause of massive hemorrhage was TURP: 17 patients (17/49, 34.7%) had received TURP before hospital admission. Of the 17 patients who had a history of TURP, eight patients (8/17, 47.1%) had received an emergency transurethral procedure one to two times to control bleeding and to clear blood clots before being transferred to Third Xiangya Hospital. Six patients (6/17, 35.3%) had suffered from hemorrhagic shock before the surgical procedure, with one of them being assisted by mechanical ventilation for pulmonary dysfunction before hospital admission. Other causes included inappropriate transurethral actions (e.g., repeatedly violent catheterization; 13/49, 26.5%), cystoscopy (10/49, 20.4%), and urethral dilatation (9/49, 18.4%).

**Table 1:** Perioperative characteristics of the patients with BMH (median, minima to maxima).

	**Urethral dilatation**	**Catheterization**	**Cystoscopy**	**TURP**
Patients number (*n* = 49)	9	13	10	17
Age (year)	72 (66–78)	72 (65–82)	71 (65–79)	74 (67–80)
PV (ml)	84 (62–87)	79 (66–107)	82 (61–97)	60 (49–73)
IPSS	33 (28–35)	30 (29–35)	29 (27–36)	30 (28–33)
QoL	4 (3–5)	4 (3–5)	4 (3–5)	4 (4–5)
PSA (ng ml^−1^)	2.6 (1.1–3.5)	2.5 (1.7–3.5)	2.4 (1.5–3.1)	2.7 (2.2–3.6)
Bleeding site				
*Apex lobe*	0	0	0	7
*Lateral lobe*	2	4	3	2
*Top lobe*^#^	0	0	0	8
*Mid lobe*	7	9	7	0
Operative time (min)	51 (41–75)	56 (41–64)	52 (43–59)	31 (25–42)
*Enucleation time*	25 (17–33)	25 (18–32)	23 (18–27)	17 (14–20)
*Resection time*	26 (21–42)	27 (20–39)	28 (25–34)	14 (9–24)
SBP (mmHg)				
*Pre-operation*	109 (100–121)^*^	106 (97–123)^*^	110.5 (108–123)^*^	95 (86–108)
*Post-operation*	123 (117–134)	130 (114–141)	121 (111–135)	130 (116–139)
Preoperative hemoglobin (g l^−1^)	99 (91–107)	97 (87–110)	101.5 (96–108)	88 (84–95)^*^
Decrease in hemoglobin (g dl^−1^)	1.5 (0.6–2.2)	1.3 (0.6–2.8)	1.6 (1–2.5)	1.6 (1.1–2.5)
Decrease in sodium (mmol l^−1^)	0.3 (0.2–0.5)	0.4 (0.2–0.8)	0.4 (0.2–1.5)	0.4 (0.2–0.8)
Resected tissue weight (g)	34 (26–39)	34 (24–45)	35.5 (26–45)	27 (19–34)
Blood transfusion	0	0	0	1
*Pre-operation*	1	0	2	10
*During operation*	0	0	0	1
*Post-operation*	0	0	0	0
Irrigation time (hour)	3 (2–7)	4 (2–7)	3.5 (2–6)	4 (2–5)
Hospitalization (day)	6 (6–7)	6 (6–7)	6 (6–7)	6 (5–6)

BMH, benign prostatic hyperplasia-associated massive hemorrhage; IPSS, international prostate symptom score; PSA, prostate-specific antigen; QoL, quality of life; SBP, systolic blood pressure; TRUS, transrectal ultrasonography; UTI, urinary tract infection; VUR, vesicoureteral reflux; PV, prostate volume; Mann–Whitney U test was used to analyze the differences between the TURP group and urethral dilatation, catheterization or the cystoscopy group with ^*^denoting p < 0.05;

# *Top lobe is located in the 12 o'clock position*.

The systolic blood pressure upon hospital admission was significantly lower in the patients who had ever received TURP (median 95 mmHg, *p* < 0.05) than that for other patients. Anemia was much more severe in the patients who had a TURP history (hemoglobin: median 88 g/L, *p* < 0.05), and 10 (10/17, 58.8%) of these patients required an infusion of homotypic concentrated erythrocyte suspension before the surgical procedure, whereas only three (3/32, 9.4%) patients without a TURP history needed a transfusion.

### Bleeding Locations

The bleeding site was demonstrated to be from the prostatic urethra intraoperatively. The patients without a TURP history commonly had edema, hyperemia, and ulceration in the prostatic urethral mucosa, along with dilated submucosal veins. Parts of the urethral mucosa or urethral glands were injured, which were mainly located in the mid lobe (23/32, 71.9%) and lateral lobes (9/32, 28.1%). Edema was present in the prostatic urethral mucosa of all the patients with a TURP history. The wound surface of these patients was uneven and, in general, had active bleeding, located mainly at the 12 o'clock position (8/17, 47.1%) and apex of the prostate gland (7/17, 41.1%), followed by the lateral urethral glands (2/17, 11.8%) (Table 1).

### Intraoperative Situation

i-TUPKEP was achieved for all the patients. In the patients with a TURP history, several residual hyperplastic glands were retained, and the surgical capsule could be identified readily from the verumontanum position for enucleation.

The surgical procedure was completed with stable vital signs and without transurethral resection syndrome for all the patients. Only one (1/49, 2%) patient needed a blood transfusion intraoperatively, and no patient (0/49) needed a blood transfusion postoperatively. Also, bleeding was controlled effectively in all the patients (Table 1).

### Follow-Up

All the patients completed the first follow-up 3 months after the surgical procedure. Also, 91.8% (45/49) of the patients returned to Third Xiangya Hospital for review 6 and 12 months after the surgical procedure, whereas four (4/49, 8.2%) patients were lost to follow-up. The IPSS and QoL score improved significantly compared with that before surgery (*p* < 0.05). During 12-month follow-up, the IPSS and QoL score remained relatively stable. The mean value of Q_max_ was close to normal, and the PVR was within the physiological range (Table 2).

**Table 2:** Follow-up data stratified by the causes of BMH (median, minima to maxima).

**Parameter**	**Urethral dilatation**	**Catheterization**	**Cystoscopy**	**TURP**
Patients number	9	13	10	17
(*n* = 49)				
IPSS
*Baseline*	31 (29–35)	31 (29–35)	33 (29–35)	30 (28–33)
*3 months*	14 (13–19)^*^	14 (10–19)^*^	17 (13–19)^*^	14 (11–17)^*^
*6 months*	12 (8–16)^*^	12 (8–16)^*^	15 (8–17)^*^	13 (10–16)^*^
*12 months*	14 (10–16)^*^	15 (11–15)^*^	13 (9–16)^*^	12 (10–15)^*^
QoL
*Baseline*	4 (3–5)	4 (3–5)	4 (4–5)	4 (4–5)
*3 months*	3 (2–3)^*^	2 (2–3)^*^	3 (2–3)^*^	2 (2–3)^*^
*6 months*	2 (2–3)^*^	2 (2–3)^*^	2 (2–3)^*^	2 (2–2)^*^
*12 months*	2 (2–2)^*^	2 (2–3)^*^	2 (2–2)^*^	2 (2–3)^*^
Qmax (ml s^−1^)
*3 months*	12 (10–16)	13 (11–16)	14 (10–15)	15 (12–16)
*6 months*	12 (11–14)	12 (10–16)	13 (11–14)	14 (12–16)
*12 months*	13 (12–15)	14 (12–15)	13 (12–15)	14 (12–15)
PVR (ml)
*3 months*	28 (27–34)	27 (24–31)	29 (27–34)	27 (23–34)
*6 months*	29 (27–30)	25 (25–30)	29 (27–30)	27 (25–30)
*12 months*	28 (25–29)	25 (21–28)	27 (25–29)	25 (23–30)

Qmax, maximum flow rate; PVR, post-void residual urine volume; Wilcoxon signed-rank test was used to analyze the differences between the baseline and 3 months, 6 months, or 12 months with ^*^denoting p < 0.05.

### Treatment-Related Adverse Events

Dysuria was identified in two patients (2/49, 4.1%) after the first time the catheter was extracted. The catheter was inserted again, and antibiotic therapy was used for 1 week. Two patients could urinate by themselves after extubation again. Seven cases (7/49, 14.3%) were complicated with a urinary-tract infection within 2 weeks of completion of the surgical procedure, and nine cases (9/49, 18.4%) had irritation or pain within the urinary tract, but relief was obtained after receiving symptomatic treatments. Only one patient (1/49, 2%) with a TURP history suffered from transient urinary incontinence, but his symptoms resolved within 2 weeks with daily training of the levator ani muscle. Anterior urethral strictures occurred 3 months after the surgery for three patients (3/49, 6.1%). The optimal treatment method was regular urethral dilation after anesthesia of the urethral mucosal surface using 2% lidocaine. Recurrent active hematuria, secondary operation, or death was not recorded (Table 3).

**Table 3:** Treatment-related adverse events.

**AEs**	**Urethral dilatation**	**Catheterization**	**Cystoscopy**	**TURP**
Patients number (*n* = 49)	9	13	10	17
Dysuria (Clavien-Dindo Grade I for all)	1	0	1	0
Urinary tract irritation/pain	2	1	2	4
*Clavien-Dindo Grade I*	2	0	1	1
*Clavien-Dindo Grade II*	0	1	1	3
UTI	1	2	1	3
*Clavien-Dindo Grade I*	0	1	0	2
*Clavien-Dindo Grade II*	1	1	1	1
TUI (Clavien-Dindo Grade I for all)	0	0	0	1
US (Clavien-Dindo Grade III for all)	0	0	1	2

*AEs, adverse events; UTI, urinary tract infection; TUI, temporary urinary incontinence; US, urethral stricture*.

## Discussion

Several aggressive transurethral actions [e.g., catheterization (15), urethra dilation (16), and cystoscopy (17)] can injure the mucosa, submucosal vessels, and, in particular, the hyperplastic mid lobe of the prostate gland, which may result in formation of a false passage and bleeding. We observed the mid lobe to be the most common bleeding site in the patients without a TURP history.

Massive hemorrhage is a severe complication of TURP (5, 18). In our study, the apex (7/17, 41.2%) and top lobe (12 o'clock position, 8/17, 47.1%) were the most common bleeding sites in the patients with a TURP history. For inexperienced surgeons, incomplete electrocoagulation on the uneven wound surface resulting from incomplete resection of hyperplastic glands, especially at sites of a relatively blinded area of the resectoscope (e.g., the 12 o'clock position of glands), may lead to severe bleeding. Moreover, to protect the external sphincter, some surgeons do not undertake efficient hemostasis at the apex of the prostatic urethra (which was also a common bleeding site in the patients in the present study). We observed that it was more urgent to deal with BMH secondary to TURP.

In addition to systemic support, the basic principles of BMH treatment are: (i) removing blood clots from the bladder; (ii) controlling bleeding; (iii) keeping the bladder drainage unobstructed (of which controlling bleeding is the core procedure). The means of hemostasis are compression of the bladder neck by a catheter balloon (19), transurethral surgery (5), and open surgery (20). Furthermore, if the patient suffers excessive loss of blood and cannot tolerate anesthesia or surgery, selective prostatic artery embolization (PAE) may be considered (21, 22). However, compression of the bladder neck and PAE cannot be employed to remove blood clots and relieve obstruction of the bladder outlet, and the hemostatic effect of these actions is uncertain. Open surgery is not first-line treatment if minimally invasive surgery is possible and safe. Therefore, if the condition permits, transurethral surgery is an ideal option for BMH because it meets all of these three principles stated above and elicits less trauma.

TURKEP combines the advantages of transurethral surgery, open prostatectomy, and a bipolar plasma kinetic system. TUPKEP is characterized by complete hemostasis, little bleeding, safety, and effective relief of bladder-outlet obstruction (10, 23, 24). During TUPKEP, a relatively loosened gap between the peripheral (surgical “capsule”) zone and the transitional zone can be exposed readily by blunt dissection (25). The surface of the capsule is smooth and characterized by a pale color and clear supply vessels. In our i-TUPKEP procedure for the patients with a TURP history, we could obtain access to the capsule and carry out enucleation. The residual glands after the previous TURP procedure can be utilized as indicators to expose the capsule.

Our single-center experience indicated that complete enucleation of proliferative glands with active bleeding and coagulation on the capsule surface could achieve effective hemostasis from the source. The bipolar plasma kinetic system can produce localized high-frequency energy, which passes through the conductive irrigation solution (0.9% saline) to tissue. In the cutting mode, if energy is sufficient, the solution is converted into a vapor layer with energy-charged particles, which touch the tissue to be resected directly, and cause its disintegration with low thermal damage to surrounding tissue. In the coagulation mode, a locally limited energy field with high power density is produced to coagulate bleeding vessels at the tissue surface. The energy is focused without being wasted on deeper tissue layers, which is efficient and safe. Owing to these traits of the system, during i-TUPKEP, the view of the surgical field upon the capsule surface is clear without damage after coagulation. Furthermore, after enucleation, electrosurgical excision of ischemic glands becomes safer and more rapid. Postoperatively, owing to improvement of contraction of the surgical capsule, the risk of re-bleeding on the wound surface is decreased markedly (26).

None of our patients suffered from active bleeding again after i-TUPKEP. The relief of symptoms due to BPH after i-TUPKEP was also satisfactory. During 12-month follow-up, the subjective indicators (the IPSS and the QoL score) of LUTS were improved, and the objective indicators (Q_max_ and PVR) were in the physiological range. However, the patients completed the questionnaires in a state of pain, which could have caused a bias in the rating process. In the future studies, more indicative parameters (e.g., the pain score) should be used for correction and evaluation.

Transient incontinence is a common complication associated with TUPKEP (27). During the surgical procedures, we carried out a small part of glands at the 12 o'clock position of the capsule and was kept as an “isolation pad.” The latter can protect the urethral sphincter from thermal damage during electrocoagulation. In our study, only one patient suffered from temporary incontinence. Infection is another common adverse event during transurethral surgery. Postoperatively, especially for the patients with small glands, inflammatory stenosis is prevalent in the prostatic urethra, which can result in dysuria after extubation (28, 29). Two cases in this setting were observed in our study, but, after placement of an indwelling catheter for 1 week and infection control, the urination symptoms improved.

Our study had five main limitations. First, our study had a retrospective design. Second, this was a single-center study. Third, the follow-up period was short (12 months). Fourth, the study cohort was small (49 cases). Fifth, i-TUPKEP was first-line treatment for BMH in our institute during the study period. Hence, there was no other method (e.g., TURP) for comparison with i-TUPKEP.

Prospective controlled studies with long-term follow-up can provide a more detailed description of the efficacy and safety of i-TUPKEP and aid exploration of the best treatment method for BMH.

## Conclusions

This was a preliminary study. Nevertheless, if preparation is satisfactory, i-TUPKEP can be employed to treat patients with BPH with BMH. Ideally, i-TURKEP should be carried out by a surgeon with experience in TUPKEP.

## Data Availability Statement

The original contributions presented in the study are included in the article/supplementary material, further inquiries can be directed to the corresponding author.

## Ethics Statement

The studies involving human participants were reviewed and approved by the Ethics Review Board of Third Xiangya Hospital at Central South University (Changsha, China). The patients/participants provided their written informed consent to participate in this study.

## Author Contributions

ZL, LH, and YG designed the research. LD, QH, and CL reviewed the medical records and collected the data. ZL and YG analyzed the data and wrote the manuscript. All authors contributed to the article and approved the submitted version.

## Funding

This project is supported by the National Natural Science Foundations of China (81902606) and the Natural Science Foundations of Hunan province (2020JJ5891) to YG.

## Conflict of Interest

The authors declare that the research was conducted in the absence of any commercial or financial relationships that could be construed as a potential conflict of interest.

## Publisher's Note

All claims expressed in this article are solely those of the authors and do not necessarily represent those of their affiliated organizations, or those of the publisher, the editors and the reviewers. Any product that may be evaluated in this article, or claim that may be made by its manufacturer, is not guaranteed or endorsed by the publisher.

## References

[1] Ayyagari R Powell T Staib L Chapiro J Perez-Lozada JC Bhatia S. Prostatic artery embolization in nonindex benign prostatic hyperplasia patients: single-center outcomes for urinary retention and gross prostatic hematuria. Urology. (2020) 136:212–7. 10.1016/j.urology.2019.11.003 31734349

[2] Zhang H Frendl DM Wang Z Olumi AF. High real-world medication adherence and durable clinical benefit in medicare patients treated with 5-alpha reductase inhibitors for benign prostatic hyperplasia. J Urol. (2020) 204:325–31. 10.1097/JU.0000000000001014 32167867 PMC8008223

[3] Bansal A Arora A. transurethral resection of prostate and bleeding: a prospective, randomized, double-blind placebo-controlled trial to see the efficacy of short-term use of finasteride and dutasteride on operative blood loss and prostatic microvessel density. J Endourol. (2017) 31:910–7. 10.1089/end.2016.0696 28650680

[4] Liu Z Li YW Wu WR Lu Q. Long-term clinical efficacy and safety profile of transurethral resection of prostate versus plasmakinetic resection of the prostate for benign prostatic hyperplasia. Urology. (2017) 103:198–203. 10.1016/j.urology.2017.02.006 28188760

[5] Cornu JN Ahyai S Bachmann A de la Rosette J Gilling P Gratzke C. A systematic review and meta-analysis of functional outcomes and complications following transurethral procedures for lower urinary tract symptoms resulting from benign prostatic obstruction: an update. Eur Urol. (2015) 67:1066–96. 10.1016/j.eururo.2014.06.017 24972732

[6] Mamoulakis C Efthimiou I Kazoulis S Christoulakis I Sofras F. The modified Clavien classification system: a standardized platform for reporting complications in transurethral resection of the prostate. World J Urol. (2011) 29:205–10. 10.1007/s00345-010-0566-y 20461386 PMC3062770

[7] Hoekstra RJ Van Melick HH Kok ET Ruud Bosch JL. A 10-year follow-up after transurethral resection of the prostate, contact laser prostatectomy and electrovaporization in men with benign prostatic hyperplasia; long-term results of a randomized controlled trial. BJU Int. (2010) 106:822–6. 10.1111/j.1464-410X.2010.09229.x 20184573

[8] Elshal AM Soltan M El-Tabey NA Laymon M Nabeeh A. Randomised trial of bipolar resection vs holmium laser enucleation vs Greenlight laser vapo-enucleation of the prostate for treatment of large benign prostate obstruction: 3-years outcomes. BJU Int. (2020) 126:731–8. 10.1111/bju.15161 32633020

[9] Welk B Reid J Ordon M Razvi H Campbell J. Population-based assessment of re-treatment and healthcare utilisation after photoselective vaporisation of the prostate or electrosurgical transurethral resection of the prostate. BJU Int. (2019) 124:1047–54. 10.1111/bju.14891 31389161

[10] Liu C Zheng S Li H Xu K. Transurethral enucleation and resection of prostate in patients with benign prostatic hyperplasia by plasma kinetics. J Urol. (2010) 184:2440–5. 10.1016/j.juro.2010.08.037 20952005

[11] Geavlete B Stanescu F Iacoboaie C Geavlete P. Bipolar plasma enucleation of the prostate vs open prostatectomy in large benign prostatic hyperplasia cases - a medium term, prospective, randomized comparison. BJU Int. (2013) 111:793–803. 10.1111/j.1464-410X.2012.11730.x 23469933

[12] David RA Badmus TA Salako AA Asaleye CM Adeloye D Fanimi O. Diagnostic performance of transrectal ultrasound for prostate volume estimation in men with benign prostate hyperplasia. Int J Clin Pract. (2020) 74:e13615. 10.1111/ijcp.13615 32683766

[13] Xu A Zou Y Li B Liu C Zheng S Li H. A randomized trial comparing diode laser enucleation of the prostate with plasmakinetic enucleation and resection of the prostate for the treatment of benign prostatic hyperplasia. J Endourol. (2013) 27:1254–60. 10.1089/end.2013.0107 23879477

[14] Mitropoulos D Artibani W Biyani CS Bjerggaard Jensen J Roupret M Truss M. Validation of the Clavien-Dindo grading system in urology by the european association of urology guidelines *ad hoc* panel. Eur Urol Focus. (2018) 4:608–13. 10.1016/j.euf.2017.02.014 28753862

[15] Willette PA Banks K Shaffer L. Visually guided male urinary catheterization: a feasibility study. J Emerg Nurs. (2013) 39:27–32. 10.1016/j.jen.2011.07.009 21937096

[16] Kim S Hosoya K Takagi S Okumura M. Outcomes following balloon dilation for management of urethral obstruction secondary to urothelial carcinoma in dogs: 12 cases (2010-2015). J Am Vet Med Assoc. (2019) 255:330–5. 10.2460/javma.255.3.330 31298635

[17] Cadish LA Ridgeway BM Shepherd JP. Cystoscopy at the time of benign hysterectomy: a decision analysis. Am J Obstet Gynecol. (2019) 220:369 e361–9 e367. 10.1016/j.ajog.2019.01.217 30685289

[18] Kavanagh LE Jack GS Lawrentschuk N. Medscape: Prevention and management of TURP-related hemorrhage. Nat Rev Urol. (2011) 8:504–14. 10.1038/nrurol.2011.106 21844906

[19] Desai M Bidair M Bhojani N Trainer A Arther A Kramolowsky E. WATER II (80-150 ml) procedural outcomes. BJU Int. (2019) 123:106–12. 10.1111/bju.14360 29694702

[20] Mireku-Boateng AO Jackson AG. Prostate fossa packing: a simple, quick and effective method of achieving hemostasis in suprapubic prostatectomy. Urol Int. (2005) 74:180–2. 10.1159/000083291 15756072

[21] Pereira K Halpern JA McClure TD Lewis NA Kably I Bhatia S. Role of prostate artery embolization in the management of refractory haematuria of prostatic origin. BJU Int. (2016) 118:359–65. 10.1111/bju.13524 27153766

[22] Kably I Pereira K Chong W Bhatia S. Prostate artery embolization (PAE) in the management of refractory hematuria of prostatic origin secondary to iatrogenic urological trauma: a safe and effective technique. Urology. (2016) 88:218–21. 10.1016/j.urology.2015.10.025 26610676

[23] Zhao Z Zeng G Zhong W Mai Z Zeng S Tao X. A prospective, randomised trial comparing plasmakinetic enucleation to standard transurethral resection of the prostate for symptomatic benign prostatic hyperplasia: three-year follow-up results. Eur Urol. (2010) 58:752–8. 10.1016/j.eururo.2010.08.026 20800340

[24] Chen YB Chen Q Wang Z Peng YB Ma LM Zheng DC. A prospective, randomized clinical trial comparing plasmakinetic resection of the prostate with holmium laser enucleation of the prostate based on a 2-year followup. J Urol. (2013) 189:217–22. 10.1016/j.juro.2012.08.087 23174256

[25] Villers A Flamand V Arquimedes RC Puech P Haber GP Desai MM. Robot-assisted partial prostatectomy for anterior prostate cancer: a step-by-step guide. BJU Int. (2017) 119:968–74. 10.1111/bju.13785 28111893 PMC9084536

[26] Peng B Wang GC Zheng JH Xia SQ Geng J Che JP. A comparative study of thulium laser resection of the prostate and bipolar transurethral plasmakinetic prostatectomy for treating benign prostatic hyperplasia. BJU Int. (2013) 111:633–7. 10.1111/j.1464-410X.2012.11610.x 23107074

[27] Huang SW Tsai CY Tseng CS Shih MC Yeh YC Chien KL. Comparative efficacy and safety of new surgical treatments for benign prostatic hyperplasia: systematic review and network meta-analysis. BMJ. (2019) 367:l5919. 10.1136/bmj.l5919 31727627 PMC7223639

[28] Reich O Schlenker B Gratzke C Tilki D Riecken M Stief C. Plasma vaporisation of the prostate: initial clinical results. Eur Urol. (2010) 57:693–7. 10.1016/j.eururo.2009.05.031 19482414

[29] Elshal AM El-Nahas AR Ghazy M Nabeeh H Laymon M Soltan M. Low-power vs high-power holmium laser enucleation of the prostate: critical assessment through randomized trial. Urology. (2018) 121:58–65. 10.1016/j.urology.2018.07.010 30031005

